# Expression of Ribonucleotide Reductase Subunit-2 and Thymidylate Synthase Correlates with Poor Prognosis in Patients with Resected Stages I–III Non-Small Cell Lung Cancer

**DOI:** 10.1155/2015/302649

**Published:** 2015-11-17

**Authors:** Francesco Grossi, Maria Giovanna Dal Bello, Sandra Salvi, Roberto Puzone, Ulrich Pfeffer, Vincenzo Fontana, Angela Alama, Erika Rijavec, Giulia Barletta, Carlo Genova, Claudio Sini, Giovanni Battista Ratto, Mario Taviani, Mauro Truini, Domenico Franco Merlo

**Affiliations:** ^1^Lung Cancer Unit, IRCCS A.O.U San Martino IST-Istituto Nazionale per la Ricerca sul Cancro, Largo Rosanna Benzi 10, 16132 Genova, Italy; ^2^Pathology and Cytohistology Division, IRCCS A.O.U San Martino IST-Istituto Nazionale per la Ricerca sul Cancro, Largo Rosanna Benzi 10, 16132 Genova, Italy; ^3^Clinical Epidemiology Division, IRCCS A.O.U San Martino IST-Istituto Nazionale per la Ricerca sul Cancro, Largo Rosanna Benzi 10, 16132 Genova, Italy; ^4^Integrated Molecular Pathology Division, IRCCS A.O.U San Martino IST-Istituto Nazionale per la Ricerca sul Cancro, Largo Rosanna Benzi 10, 16132 Genova, Italy; ^5^Epidemiology, Biostatistics and Clinical Trials Division, IRCCS A.O.U San Martino IST-Istituto Nazionale per la Ricerca sul Cancro, Largo Rosanna Benzi 10, 16132 Genova, Italy; ^6^Thoracic Surgery Division, IRCCS A.O.U San Martino IST-Istituto Nazionale per la Ricerca sul Cancro, Largo Rosanna Benzi 10, 16132 Genova, Italy

## Abstract

Biomarkers can help to identify patients with early-stages or locally advanced non-small cell lung cancer (NSCLC) who have high risk of relapse and poor prognosis. To correlate the expression of seven biomarkers involved in DNA synthesis and repair and in cell division with clinical outcome, we consecutively collected 82 tumour tissues from radically resected NSCLC patients. The following biomarkers were investigated using IHC and qRT-PCR: excision repair cross-complementation group 1 (ERCC1), breast cancer 1 (BRCA1), ribonucleotide reductase subunits M1 and M2 (RRM1 and RRM2), subunit p53R2, thymidylate synthase (TS), and class III *beta*-tubulin (TUBB3). Gene expression levels were also validated in an available NSCLC microarray dataset. Multivariate analysis identified the protein overexpression of RRM2 and TS as independent prognostic factors of shorter overall survival (OS). Kaplan-Meier analysis showed a trend in shorter OS for patients with RRM2, TS, and ERCC1, BRCA1 overexpressed tumours. For all of the biomarkers except TUBB3, the OS trends relative to the gene expression levels were in agreement with those relative to the protein expression levels. The NSCLC microarray dataset showed RRM2 and TS as biomarkers significantly associated with OS. This study suggests that high expression levels of RRM2 and TS might be negative prognostic factors for resected NSCLC patients.

## 1. Introduction

Only 30–40% of new patients diagnosed with NSCLC have disease confined to the thorax. The standard of care for patients with early-stage NSCLC is surgical resection, but 50–60% of patients with local disease relapse within two years [1].

The 5-year overall survival (OS) by pathologic stage is, respectively, 73% and 54% for stages IA and IB, 48% and 38% for stages IIA and IIB, and 25% and 19% for stages IIIA and IIIB [2]. Because of this high and rapid recurrence rate, adjuvant chemotherapy after surgery is recommended for selected stages IB, II, and III patients [3, 4].

The only criterion currently used in standard practice to estimate prognosis is disease stage. Therefore, the discovery of prognostic markers that are different and independent of tumour stage represents a high medical need that has been unmet thus far.

In this study, we evaluated the protein and mRNA expression levels of seven biomarkers involved in DNA repair (ERCC1, BRCA1, RRM1, RRM2, and p53R2), DNA synthesis (TS), and cellular division (TUBB3) to test the hypothesis that these biomarkers could act as prognostic factors in radically resected NSCLC patients. We also investigated the association of gene expression with OS using a large publicly available NSCLC microarray dataset [5].

Briefly, ERCC1 is a crucial component of the nucleotide excision repair (NER) pathway that repairs DNA damage following exposure to platinum agents. The IALT-Bio study, using an IHC-based H-scoring system, showed that, in patients who did not receive adjuvant chemotherapy, the 5-year survival rate among ERCC1-positive patients was higher (46%) compared to ERCC1-negative patients (39%) (*p* = 0.009), demonstrating that ERCC1 expression levels may be a valuable indicator of prognosis [6]. Subsequently, the same authors [7] investigated the tumour specimens from the IALT-Bio study for ERCC1 expression using a fluorescence-based automated scoring system (AQUA). The difference in OS for patients with high and low ERCC1 expression had a similar trend as that previously reported with IHC in the control group (untreated patients), although the difference was not statistically significant. These conflicting findings place doubts on the role of ERCC1 in resected NSCLC patients' outcome justifying further investigations.

BRCA1 has multiple roles not only in DNA damage repair but also in cell cycle regulation, transcriptional control, ubiquitination, and apoptosis [8]. Rosell et al. [9] evaluated the association between BRCA1 mRNA expression and survival in radically resected NSCLC patients and demonstrated that high expression levels were strongly associated with poor survival.

Another important molecule involved in DNA synthesis and repair is the enzyme ribonucleotide reductase (RR) that catalyses the conversion of ribonucleotides into deoxyribonucleotides [10]. RR consists of three subunits, RRM1, RRM2, and p53R2. RRM1 contains enzymatically active sites and binding sites for allosteric effectors. The p53R2 gene contains a p53-binding sequence and can functionally substitute for RRM2 because the genes are homologous (80%) and both possess a diiron-tyrosyl radical cofactor that is essential for enzyme activity [11]. RRM1 interacts with either RRM2 or p53R2 to become the catalytically active form of eukaryotic RR. The prognostic role of p53R2 in stages I–III NSCLC patients was investigated by Uramoto et al. [12] who concluded that p53R2 did not play an important prognostic role and that the pathway mediated by p53R2 may be responsible for controlling the growth of lung cancer. Conversely, Hsu et al. [13, 14] showed that the presence of p53R2 protein is a favorable prognostic factor in early-stage lung cancer. There is limited information concerning the prognostic role of RRM2 and RRM1 mRNA expression in human lung tumours. Loss of heterozygosity (LOH) for the RRM1 gene has been correlated with poor survival in resected NSCLC patients [15] and has been found to be a significant adverse prognostic factor. Preclinical studies have revealed a potential prognostic role for RRM2 demonstrating that cells that overexpress RRM2 mRNA exhibit enhanced cellular invasiveness [16] through activation of nuclear factor kB (NF-*κ*B) and increased matrix metalloproteinase-9 (MMP-9) expression [17].

Microtubules consist of *α*-tubulin and *β*-tubulin dimers and are critical for cell growth and division. The tubulins can exist in various isotypes and TUBB3 is one of the six human isotypes that comprise microtubules. Sève et al. [18, 19] conducted a retrospective study to explore the potential of this biomarker as a prognostic or predictive factor in advanced NSCLC patients and provided strong evidence that the overexpression of TUBB3 has a predictive value for paclitaxel therapy but is not itself a prognostic factor. In contrast, Reiman et al. [20] conducted a meta-analysis considering the prognostic and predictive value of TUBB3 in resected NSCLC patients enrolled in four randomized controlled trials of adjuvant chemotherapy, showing a prognostic effect of high TUBB3 expression while they were unable to demonstrate its predictive role in adjuvant setting.

Finally, TS is an essential enzyme for* de novo* DNA synthesis and DNA damage repair and is a key target for cancer chemotherapeutic agents. Higher TS mRNA expression levels have been shown in squamous cell carcinoma compared to adenocarcinomas [21]. Nakagawa et al. [22] demonstrated that TS status is a significant prognostic factor in resected adenocarcinoma of the lung suggesting that patients with high TS expression levels have poor survival. However, further evidence is needed to confirm the clinical importance of TS expression. The aim of this study was to correlate the gene and protein expression levels of these seven biomarkers with clinicopathologic features and clinical outcome of patients with resected NSCLC to investigate their possible prognostic role.

## 2. Materials and Methods

### 2.1. Study Population

Tumour samples from 82 consecutive patients with stages I–III NSCLC who had undergone surgical resection at the National Institute for Cancer Research (Genova, Italy) between July 2005 and March 2007 were examined for gene and protein expression after obtaining approval from the Institutional Review Board. The study was done in compliance with the principle of the Declaration of Helsinki and written informed consent for use of tissue was acquired from patients at the time of first outpatient visit. All tumours were curatively resected without microscopic residual tumours by lobectomy, bilobectomy, or pneumonectomy. None of the patients received adjuvant radiation or chemotherapy. Each patient's vital status was ascertained across the follow-up period of July 2005–July 2010, and survival time was computed from the date of surgery to death or the end of follow-up for patients that remained alive.

Patients and tumour characteristics are shown in Table 1.

### 2.2. Tissue Microarrays Construction and Immunohistochemistry

Tumour tissue microarrays (TMAs) and immunohistochemistry (IHC) were performed as previously reported [23, 24], using 82 Formalin Fixed Paraffin Embedded (FFPE) primary NSCLC samples. Slides of tumour samples stained with hematoxylin and eosin were independently reviewed by two pathologists (M.T. and S.S.), and representative areas were marked. Core tissue biopsy specimens (2 mm in diameter) were obtained in duplicate from each donor tumour block and placed in a new recipient paraffin block (tissue array block) with a manual tissue microarrayer (Galileo TMA CK 3500, Bio Rep, Milano). Sections (3 *μ*m) were cut from each tissue array block, placed on slides, deparaffinised, dehydrated, and used for immunohistochemical analysis. TMA IHC was performed using the automatic immunostainer Benchmark XT (Ventana Medical Systems, SA Strasbourg, France). Briefly, antigen-retrieval was performed using citrate buffer (pH 6) at 90°C for 30 minutes. Then, the slides were incubated in primary antibody for 1 hour at 37°C followed by the addition of the polymeric detection system Ventana Medical System Ultraview Universal DAB Detection Kit. Finally, the slides were counterstained with modified Gill's hematoxylin and mounted in Eukitt.

In detail, the primary antibodies used and the positive control for each biomarker were as follows:

- *BRCA1*, clone GLK-2 (Diagnostic Bio System); epitope: peptide corresponding to amino acids 1839–1863 of the c-terminus of BRCA1; species: mouse; dilution 1 : 100, visualization: nuclear, nuclear/cytoplasmatic; and positive control: ovarian carcinoma.
- *p53R2*, clone p53R2 (Novus Biological); epitope: peptide corresponding to amino acids 2–17 of p53R2; species: rabbit polyclonal; dilution 1 : 200; visualization: nuclear; and positive control: lung cancer (normal and tumoural).
- *ERCC1*, clone 8F1 (Diapath); epitope not determined; species: human and rat; dilution 1 : 100, visualization: nuclear; and positive control: lung cancer (normal and tumoural).
- *TS*, clone TS 106 (DAKO); epitope: peptide corresponding to amino acids 133–142 of TS; species: mouse; dilution 1 : 10; visualization: cytoplasmatic, nuclear/perinuclear; and positive control: tonsil and lymph node.
- *TUBB3*, clone MU 177-UC (Biogenex); epitope not available; species: mouse; dilution 1 : 100; visualization: cytoplasmatic; and positive control: lung cancer (normal and tumoural).
- *RRM1*, clone NA (Spring); epitope not available; species: rabbit; dilution 1 : 10; visualization: cytoplasmatic; and positive control: lung cancer (normal and tumoural).
- *RRM2*, clone 1E1 (Novus Biological); epitope not available; species: mouse; dilution 1 : 10; visualization: cytoplasmatic; and positive control: breast cancer (normal and tumoural).

An appropriate external positive control tissue was used for each staining procedure; the negative control consisted of performing the entire IHC procedure on an adjacent section in the absence of the primary antibody. Stained slides were analysed by two independent observers using an optical microscope (Olympus BX41) with 10x and 40x objectives. Immunoreactivity was graded in the tumours according to the number of immunoreactive cells and/or staining intensity using a scoring system. Regarding TS expression, the immune reaction was graded as negative (score 0) or positive, in a semiquantitative, 3-tier system based on the extent of reactivity (score 1, 1–10% reactivity; score 2, 11–50% reactivity; and score 3, >50% reactivity) [21]. Expression of ERCC1 was quantified using a visual grading system based on the extent of staining (percentage of tumour cells) graded on a scale of 0–3 (0 = no staining, 1 = weak staining, 2 = moderate staining, and 3 = strong staining) [25]. Expressions of BRCA1, RRM1, RRM2, p53R2, and TUBB3 were evaluated semiquantitatively based on staining intensity and proportion. The proportion of staining was scored on a scale from 0 to 3 as follows: diffuse, ≥50% positive (score 3); regional, 10–49% positive (score 2); focal, 1–9% (score 1); and negative (score 0). In addition, the staining intensity was scored from 0 to 3 (0, absent; 1, weak; 2, moderate; and 3, intense) [26]. A final histochemical score (*H*-score) for each sample was calculated as previously reported [24]. Positive staining (BRCA1, p53R2, and TS) or the median score values (ERCC1, RRM1, RRM2, and TUBB3) were used as cut-off criteria to categorise patients in two groups for statistical analysis.

**Figure 1:**
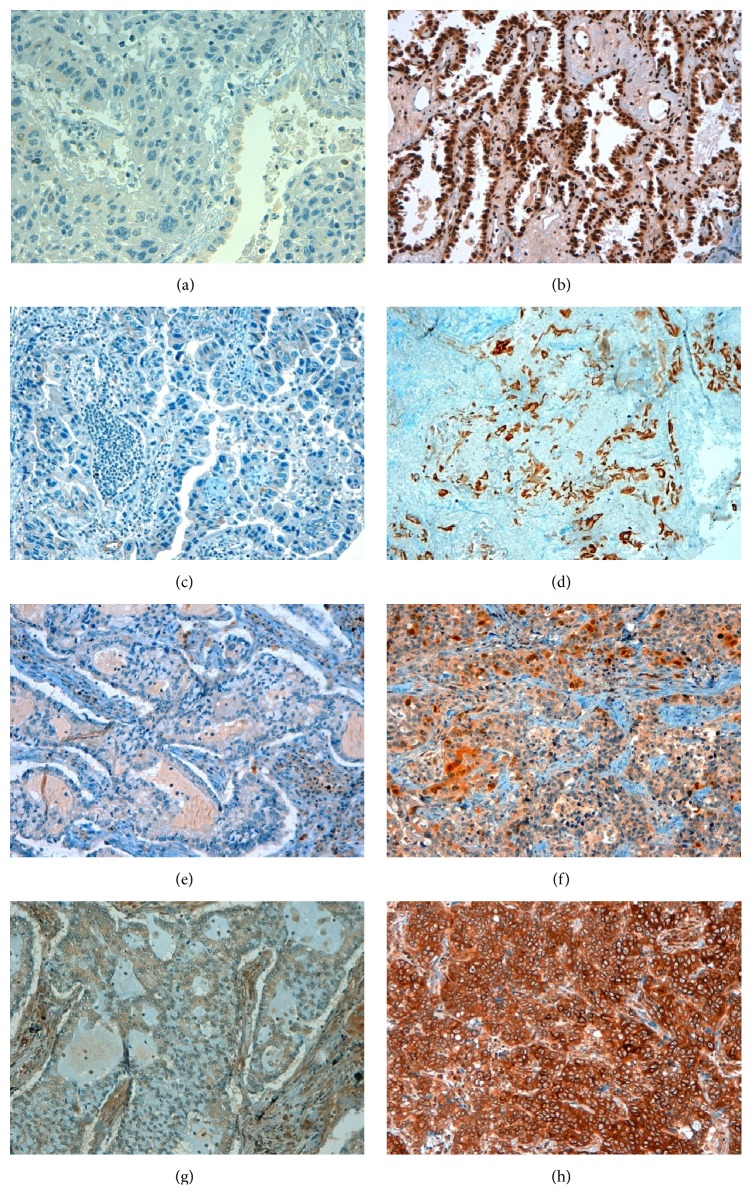
Representative results of IHC staining of NSCLC tumour specimens for ERCC1, BRCA1, TS, and TUBB3. (a) Negative expression of ERCC1 (final score 0), (b) high expression of ERCC1: nuclear staining (final score 3); (c) negative expression of BRCA1 (final score 0), (d) high expression of BRCA1: cytoplasmic staining (final score 2); (e) negative expression of TS (final score 0), (f) high expression of TS: cytoplasmic staining (final score 1) and nuclear staining (final score 2); and (g) negative expression of TUBB3 (final score 0), (h) high expression of TUBB3: cytoplasmic staining (final score 3).

**Figure 2:**
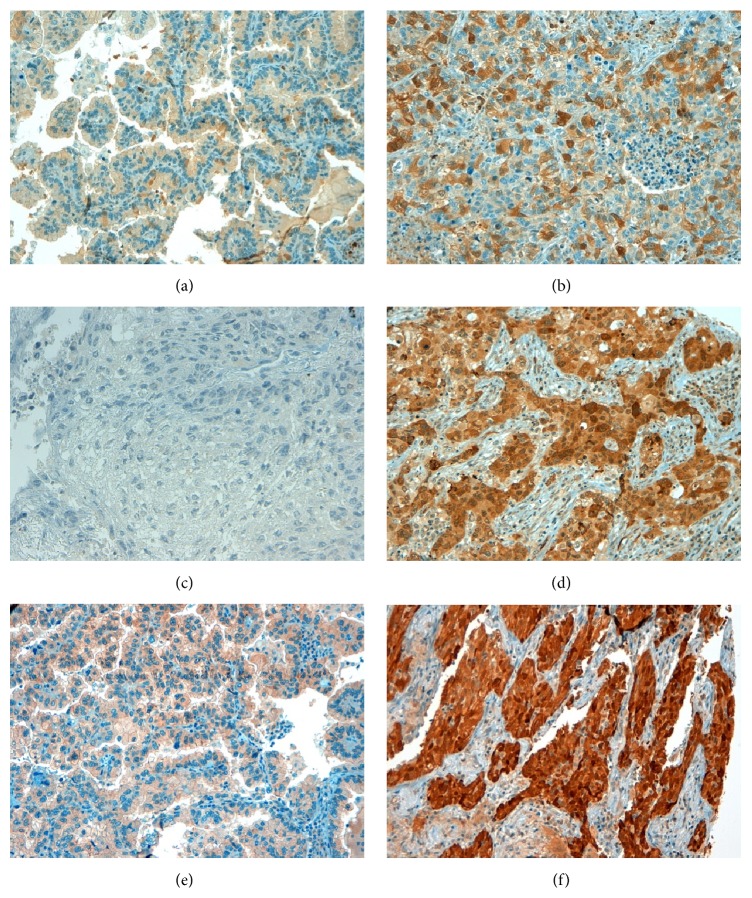
Representative results of IHC staining of NSCLC tumour specimens for RRM2, RRM1, and p53R2. (a) Negative expression of RRM2 (final score 0), (b) high expression of RRM2: cytoplasmic staining (final score 3); (c) negative expression of RRM1 (final score 0), (d) high expression of RRM1: cytoplasmic staining (final score 2); and (e) negative expression of p53R2 (final score 0), (f) high expression of p53R2: nuclear staining (final score 3).

Representative results of immunohistochemical staining of NSCLC specimens are provided in Figures 1-2.

### 2.3. Reverse Transcription and qRT-PCR

RNA was isolated from the 82 FFPE tumour samples used for the TMA construction using the High Pure FFPE RNA Micro Kit (Roche Applied Science, Mannheim, Germany) with minor modifications. For each FFPE block, a representative H&E stained section was reviewed by a pathologist to consider the tumour cells content. Whether the neoplastic elements were at least 70% of the total cell population, the tumour block was considered suitable for the analysis. From each FFPE block, four 10 *μ*m-thick sections were deparaffinised twice with 1.0 mL Histo-Clear (National Diagnostics, Atlanta, GA, USA) for 5 min at room temperature, followed by washing in 1.0 mL of 100% ethanol and 70% ethanol. The tissue pellet was air-dried for 15 min at 55°C and then lysed by incubation overnight with proteinase K at 55°C until the digestion was complete. Genomic DNA contamination was removed using an on-column DNase I treatment. RNA yield and purity were checked with a NanoDrop-1000 Detector (NanoDrop-Technologies, Wilmington, NC, USA).

After isolation, one microgram of RNA was reverse-transcribed with an engineered version of M-MLV Reverse Transcriptase (SuperScript II RT, Invitrogen, Grand Island, NY, USA) according to the manufacturer's instructions and the resulting cDNA was amplified by the LightCycler 480 Real Time PCR System II (Roche Applied Science). PCR reactions were performed in a final volume of 20 *μ*L containing 2 *μ*L of cDNA, 10 *μ*L of LightCycler 480 SYBR Green I Master Mix (Roche Applied Science), and 4 *μ*L of 2 *μ*M forward and reverse primers. The thermal profile for the samples amplification included an initial incubation at 95°C for 10 minutes for activation of FastStart Taq DNA Polymerase, 45 cycles of denaturation at 95°C for 10 seconds followed by annealing at 60°C, and extension at 72°C for 15 seconds. All the samples were amplified in triplicate with appropriate nontemplate controls. Specific forward and reverse primers were designed by Primer3 software (http://bioinfo.ut.ee/primer3-0.4.0/) on the basis of gene sequences obtained from the GenBank. All primers were intron-spanning to avoid genomic DNA contamination and the oligonucleotide sequences are presented in Table 2. The housekeeping genes* beta*-2-microglobulin (B2M) and* beta*-glucuronidase (GUSB) were used for their suitability as internal references in clinical lung cancer specimens [27, 28]. Relative gene expression levels were calculated by the 2^−ΔCt^ method (LightCycler 480 SW 1.5) and samples were normalized for the mean of the two housekeeping genes as measured by analysis with Bestkeeper software (http://www.wzw.tum.de/gene-quantification/bestkeeper.html) [29].

For each biomarker, the median of the gene expression level was used as the cut-off criteria to categorise patients in two groups for statistical analysis.

### 2.4. DCC NSCLC Microarrays Dataset Retrieval and Analysis

The DCC NSCLC dataset is a microarray data collection obtained from 442 resected tumours from NSCLC patients. Affymetrix UG133a microchip arrays were used, which contain approximately 22k probes each. The microarray “CEL” files with extensive clinical and pathological data are publicly available [https://array.nci.nih.gov/caarray/project/details.action?project.id=182]. The full dataset has been published previously [5]. For the analysis, we included 330 patients with stages I–III tumours that had not received any adjuvant therapy. Age at diagnosis, smoking status, and ERCC1, BRCA1, TS, RRM1, RRM2, and TUBB3 gene expressions were retrieved for statistical analysis (probes for p53R2 are not present in the G133a platform). Standard data processing was applied: GCRMA processing of CEL files [30], probe signal filtering, corrections for known bias [31], mean of probe signals related to the same gene, and gene-level normalisation. Patients were categorised in two groups according to the level of gene expression (i.e., ≤ and > median value of each biomarker) for statistical analysis.

### 2.5. Statistical Analysis

The associations between biomarker levels and patient and tumour characteristics were investigated by means of the Mann-Whitney *U* test. Pearson's correlation coefficient was computed to measure the relationships between protein and mRNA expression levels for each biomarker. The Kaplan-Meier (K-M) product limit estimator was used to generate survival plots and the log-rank test to compare survival distributions. To this aim, survival time was defined as the difference between date of death or date of end of follow-up, whichever came first, and date of radical surgery and was expressed in years. Cox proportional hazards multiple regression analysis of OS was performed to identify biomarkers with a significant prognostic role, adjusted for the effect of patients and tumours characteristics (i.e., age at diagnosis: ≤70 and >70 years, smoking habit: ex/never-smoker, current smoker, pathological TNM: stages I, II, and III, and histological type: nonsquamous, squamous). The stepwise backward procedure was used to select variables contributing to the Cox model as allowed by the IBM SPSS [32] statistical software. The *p* values for variable entry or removal were 0.05 and 0.10. Hazard ratio (HR) point estimates and 95% confidence interval (95% CI) were computed and differences in OS were considered statistically significant at a *p* value of <0.05. The two-sided log-rank and likelihood ratio statistics were computed to test differences between K-M survival probabilities and HRs estimated by Cox regression. All analyses were performed using IBM-SPSS statistical software, version 20, and the Bioconductor libraries [33].

## 3. Results

### 3.1. Associations between the IHC Expression of Biomarkers, Patients, and Clinicopathologic Features and Overall Survival

**Figure 3:**
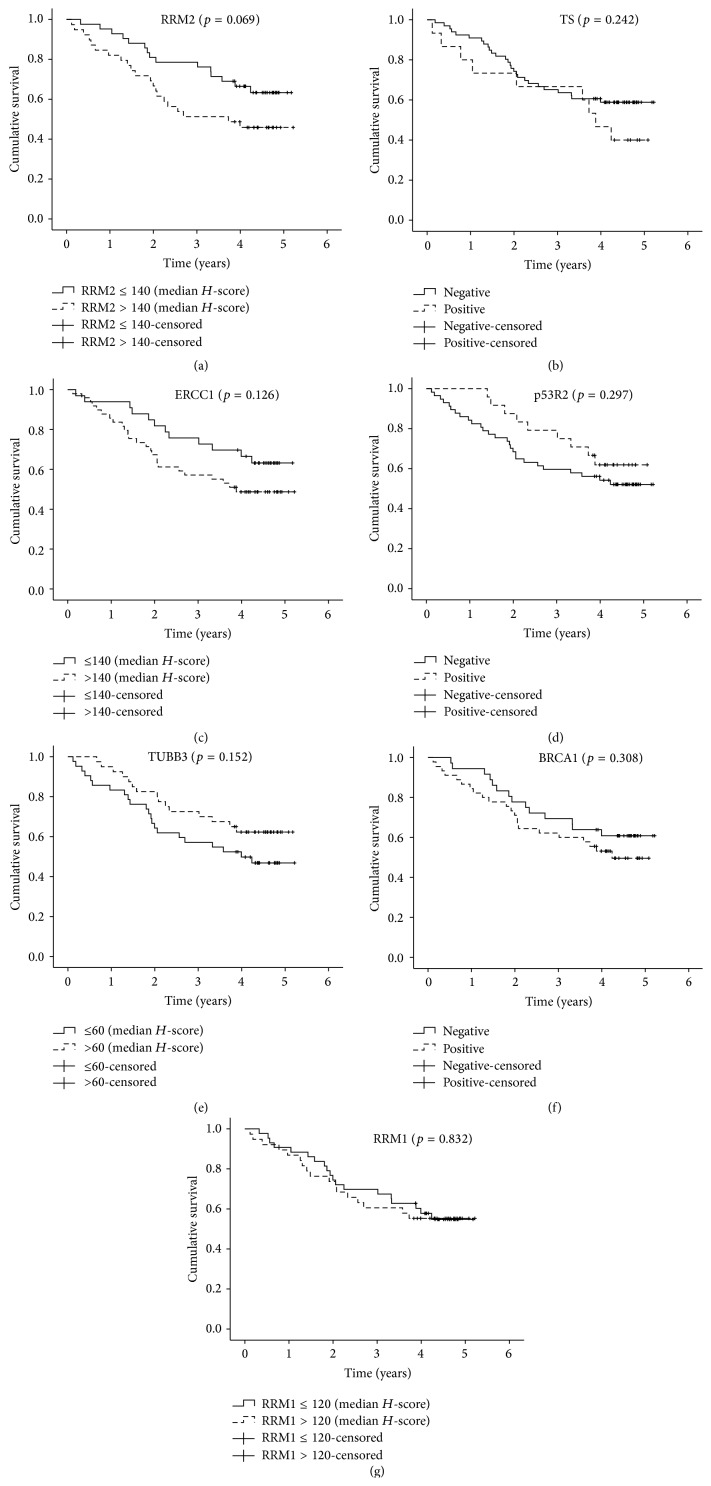
Kaplan-Meier estimates of overall survival according to the protein expression levels of (a) RRM2, (b) TS, (c) ERCC1, (d) p53R2, (e) TUBB3, (f) BRCA1, and (g) RRM1 in the overall population.

Among the investigated associations between IHC expression of biomarkers, patients, and clinicopathologic features, only subunit p53R2 was found to be significantly associated with histotype (*p* < 0.001) with more p53R2-positive cases in nonsquamous than in squamous cancer (data not shown). The associations between patient and cancer characteristics, biological markers expression, and OS are shown in Table 3. Among all patients, a total of 37 (45%) deaths were observed during follow-up. The probability of surviving at 1 year and 2, 3, 4, and 5 years was 89%, 73%, 65%, 56%, and 54%, respectively. Pathological TNM was the only variable statistically associated with OS in univariate analysis (stage II, HR = 2.81, 95% CI = 1.18–6.69; stage III, HR = 4.44, 95% CI = 2.08–9.46, *p* < 0.001) (Table 3). K-M estimated mean survival time was 4.47 years for stage I patients (median survival time not reached), 3.12 years (median 2.69 years) for stage II patients, and 3.70 years (median 1.80 years) for stage III patients (data not shown). K-M analysis showed that despite the lack of statistical significance, patients with lower RRM2 expression (i.e., ≤140) survived longer (HR = 1.84; 95% CI = 0.95–3.56) than patients with higher RRM2 expression (Table 3, Figure 3). The median survival was not reached for RRM2 ≤ 140 and was 3.7 years for RRM2 > 140. There was a trend towards longer survival for BRCA1-, ERCC1-, and TS-negative patients and for p53R2- and TUBB3-positive patients (Figure 3).

The multivariate Cox proportional hazards model (Table 3) identified pathologic stage (stage II, HR = 3.51, 95% CI = 1.35–9.07; stage III, HR = 6.54, 95% CI = 2.86–14.9, *p* < 0.001) and the overexpression of RRM2 (HR = 2.26; 95% CI = 1.08–4.74; *p* = 0.031) and TS (HR = 2.93; 95% CI = 1.16–7.42; *p* = 0.023) as independent prognostic factors for OS. Additionally, ERCC1 was found to be associated with shorter survival (HR = 2.08; 95% CI = 0.97–4.44; *p* = 0.059) but failed to reach statistical significance (*p* = 0.059). Neither RRM1, p53R2, TUBB3, and BRCA1 expression nor gender, age at diagnosis, smoking habits, and histological type were associated with OS.

### 3.2. mRNA Expression of Biological Markers and Clinical Outcome

The Mann-Whitney *U* test showed that there was no association between age, gender, pathological stage, cancer histology, or smoking habits and mRNA levels for all biomarkers analysed (data not shown). Analysis of the correlation between protein and mRNA expression levels (Table 4) showed significant correlations for RRM1 (*r* = 0.29, *p* < 0.01) and TS (*r* = 0.47, *p* < 0.05) and for TS and TUBB3 (*r* = −0.27, *p* < 0.05). Recent data consistently suggest that TUBB3 and TS expression were significantly correlated to poor outcomes in NSCLC patients; therefore, their expression could correlate to aggressive tumour behavior and increased proliferative activity [34, 35]. However, the underlying mechanism relating TUBB3 and TS expression to poor prognosis is unknown and needs to be elucidated by future studies.

**Figure 4:**
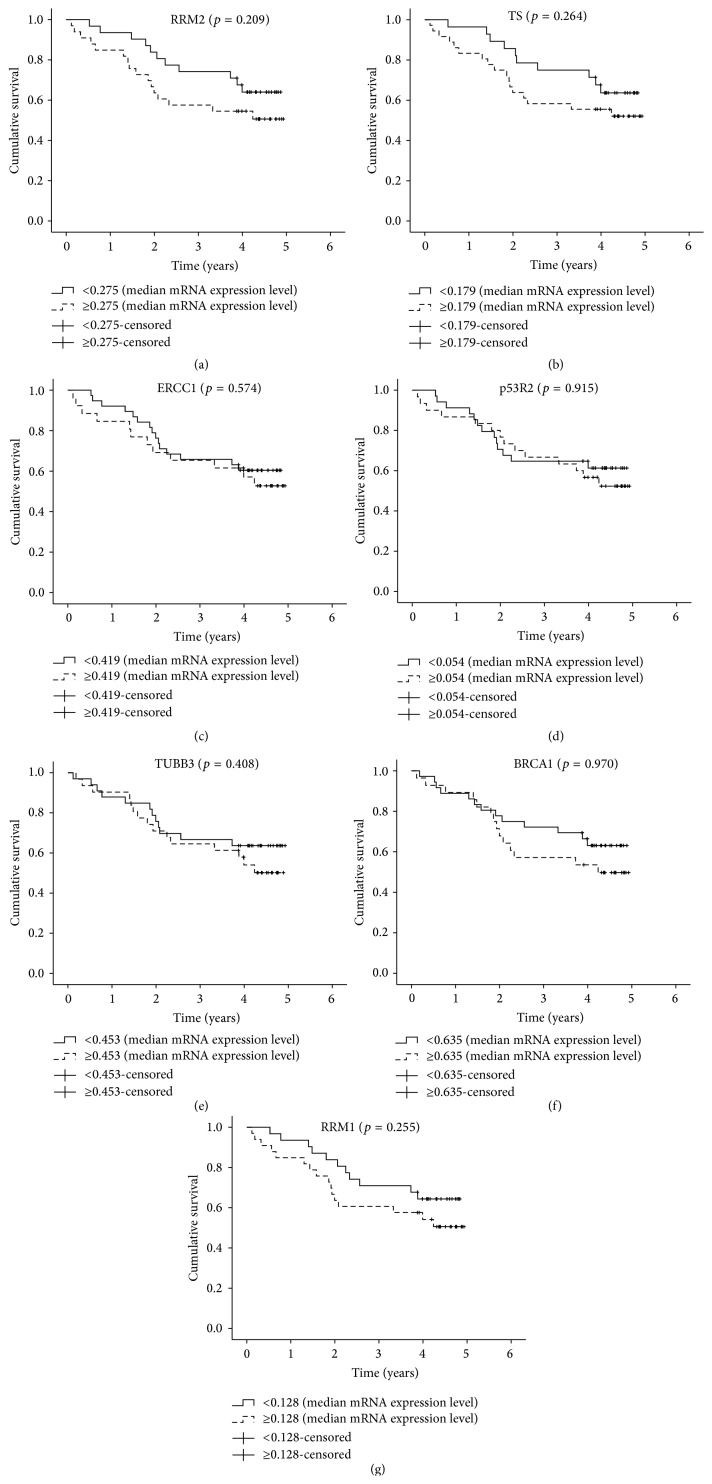
Kaplan-Meier estimates of overall survival according to the mRNA expression levels of (a) RRM2, (b) TS, (c) ERCC1, (d) p53R2, (e) TUBB3, (f) BRCA1, and (g) RRM1 in the overall population.

When patients were classified into groups based on low and high levels of biomarkers mRNA expression (Table 5), no statistically significant association with OS was detected. K-M plots were similar to those obtained for protein levels except for TUBB3 for which the trend was inverted indicating a longer OS among patients with lower mRNA expression levels (Figures 3-4). Cox multiple regression limited to 64 subjects with complete data (Table 5) identified only pathological TNM as a significant predictor of OS with worse survival associated to stages II (HR = 3.49, 95% CI = 1.30–9.40) and III (HR = 4.39, 95% CI = 1.41–10.68) compared to stage I.

### 3.3. Biomarkers' mRNA Expression in the DCC Microarray Dataset

**Figure 5:**
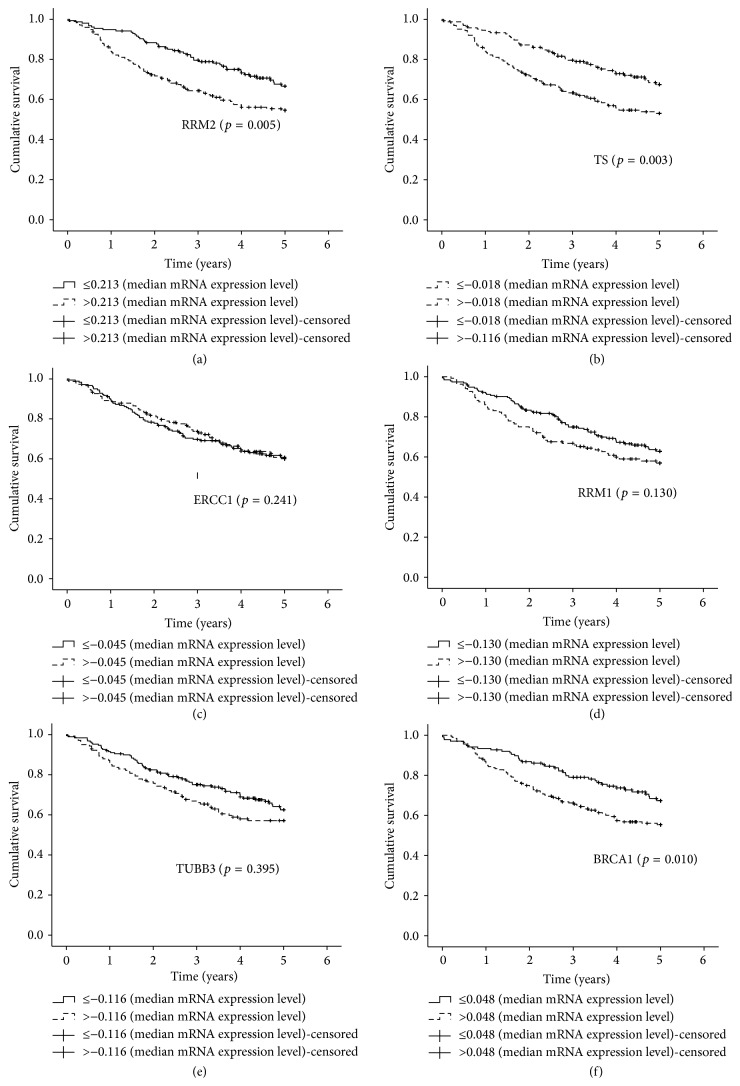
Kaplan-Meier estimates of overall survival according to gene expression levels of (a) RRM2, (b) TS, (c) ERCC1, (d) RRM1, (e) TUBB3, and (f) BRCA1 in Shedden 2008 [5] microarray dataset; *N* = 330 (adjuvant naïve patient only). Patients were categorised according to mRNA expression levels of each gene.

One hundred and seventy-four out of 330 patients were male (52.7%) and 156 (47.3%) were female. The median age at diagnosis was 65 years (range of 33–87). All of the patients had adenocarcinoma. Twenty-five (7.6%) patients were smokers, 182 (55.2%) former smokers, and 34 (10.3%) never-smokers. Smoking was missing for 89 (26.9%) subjects. Former and current smokers were grouped together in the statistical analysis (Table 6). At 5-year follow-up, a total of 121 (36.7%) deaths were observed. When patients were categorised into groups based on mRNA expression levels of each biomarker (i.e., negative, positive), three genes were found to be significantly associated with OS in univariate analyses (Table 6): BRCA1 (HR = 1.64, 95% CI = 1.12–2.39), TS (HR = 1.78, 95% CI = 1.24–2.56), and RRM2 (HR = 1.69, 95% CI = 1.17–2.44). The K-M survival curves for these genes are shown in Figure 5. Pathological TNM was a strong predictor of OS (HR = 3.19, 95% CI = 2.18–4.66 and HR = 6.45, 95% CI = 3.43–12.15 for stages II and III versus stage I, resp.) together with age at diagnosis (HR = 1.03, 95% CI = 1.01–1.04, and age = continuous) and gender (HR = 0.68, 95% CI = 0.47–0.98, females versus males). Cox multiple regression analyses (Table 6) identified age at diagnosis, pathological TNM, and TS (HR = 1.57, 95% CI: 1.08–2.28) as variables significantly associated with OS.

## 4. Discussion

We investigated the effect of the expression of seven biomarkers (ERCC1, BRAC1, RRM1, RRM2, P53R2, TUBB3, and TS) on survival in stages I–III NSCLC patients treated with surgery alone. The majority of these markers have already been widely investigated for their predictive role with the aim of customising postoperative adjuvant chemotherapy [36–38] or first line treatment [39–43]. Conversely, many prognostic molecular markers have been described for patients with NSCLC, but none are currently being used to determine the course of treatment [44–50].

Our study is the first that considers all of these markers together, using two different research methods (IHC and qRT-PCR), with the aim of testing their influence on survival and identifying patients with a higher risk of relapse.

TNM stage, widely used in standard practice to select chemotherapy drugs in the treatment of NSCLC, has been confirmed as the main prognostic factor. The need to identify new molecular markers of recurrence for determining clinical outcome and improve survival in patients with early-stage NSCLC has clearly emerged during the recent years. Our study has shown that the protein expression of RRM2 is significantly associated with OS in surgically resected NSCLC patients. In particular, in agreement with recent reports [14, 51], patients with underexpressed RRM2 tumours survived longer after radical surgery than those with overexpressed RRM2 tumours. Thus, loss of DNA repair function may be an advantage for NSCLC patients following tumour resection. Notably, RRM2 was a good prognostic indicator of OS in univariate analyses (HR = 1.84, 95% CI = 0.95–3.56) as well as in multivariate Cox regression analysis (HR = 2.26, 95% CI = 1.08–3.56) when the significant effects of pathological TNM, TS, and ERCC1 on OS were taken into account. This finding may reflect the crucial role of RRM2 in supplying deoxyribonucleotides (dNTPs) during DNA synthesis and repair and is in agreement with the observation that a high level of RRM2 expression correlates with cellular invasiveness [14, 15], tumour angiogenesis [52], and metastasis [53]. Therefore, patients with tumour cells overexpressing RRM2 may more easily progress, thus supporting our finding that RRM2 expression levels may be a valuable indicator of prognosis.

We also observed that TS protein expression was an unfavourable prognostic factors in multivariate analysis with a trend towards poor postoperative survival among patients with tumours overexpressing TS. This result is in agreement with its key role in methylation of deoxyuridine monophosphate (dUMP) to deoxythymidine monophosphate (dTMP), required for DNA synthesis and repair, and with the evidence that TS expression is significantly correlated with increased proliferative activity and aggressive tumour behaviour [54, 55]. Thus, it is realistic to assume that TS might play an important role in regulating the malignant potential in many types of cancer not only in lung cancer. No significant correlation between intratumoural TS protein and gene expression levels and clinicopathologic characteristics was observed. These findings obtained for TS were in agreement with previously reported evidence [22, 35, 56, 57], while discordant results were found for ERCC1. The relevance of ERCC1 expression as prognostic marker has been reported in some studies [6, 58] but the conflicting information regarding the pure prognostic role of ERCC1 expression remains and is also supported by our study. Previous studies [58–61] have shown that high ERCC1 levels are associated with longer survival. Conversely, our study showed that there was a tendency towards better prognosis in ERCC1-negative cases, although the difference was not statistically significant. Recent articles [62, 63] support this result showing that ERCC1 expression does not affect survival in patient who did not previously receive adjuvant chemotherapy. An additional study [9] showed that a low ERCC1 expression is associated with a significantly better prognosis in stage I NSCLC. The results of our study might be affected by our study population because the majority of our patients were stage I and did not receive any adjuvant chemotherapy. Furthermore, recent studies [64, 65] have suggested that all these conflicting results might depend on the antibody used for the detection of ERCC1. Since none of the ERCC1 antibodies usually used distinguish between functional and nonfunctional isoforms, it might be difficult to validate the correlation between the level of ERCC1 expression and OS on the basis of IHC detection; in effect, the expression of nonfunctional ERCC1 isoforms may lead to false ERCC1-positive cases and biased results.

Although in our study p53R2 did not have a significant effect on OS, it was significantly associated with histotype. There were more p53R2-positive cases in nonsquamous than in squamous NSCLC and this is concordant with a previous study [12] and with the evidence that patients with adenocarcinoma have a worse prognosis than patients with squamous cell carcinoma. We hypothesized its possible role as a marker of more aggressive tumoural phenotype since it plays an important role in the DNA repair pathway and its expression could increase following DNA damage or accumulation of several genetic changes [11]. In this sense, p53R2 may be useful for detecting aggressive tumours with high metastatic potential and a poor prognosis.

Rosell et al. [9] showed that BRCA1 mRNA expression, implicated in transcription-coupled nucleotide excision repair (TC-NER) pathway, was the only independent prognostic variable in chemotherapy-naïve patients with early-stage, resected NSCLC and demonstrated that RRM1 mRNA expression did not show a statistically significant impact on OS. The author reported that prolonged survival was observed in BRCA1- and RRM1-negative tumours but not among patients with BRCA1- and RRM1-positive tumours. According to this study, our findings, using IHC evaluation, have shown that higher expression of BRCA1 and RRM1 correlates with poorer survival in early NSCLC even if the difference between the survival curves was not statistically significant.

Moreover, a previous study has shown that class TUBB3 has a negative prognostic role in patients with curatively resected NSCLC who did not receive adjuvant chemotherapy [66]. Our study did not show a prognostic role of TUBB3 on OS in the same group of NSCLC patients. Moreover, no significant correlations between BRCA1, RRM1, and TUBB3 expression levels and clinicopathological characteristics were found. Even though the sample size of the present study is limited, and thus the study itself is underpowered to detect significant differences between biomarkers expression and OS, our results suggest an interesting trend in survival curves not only for RRM2 but also for most of the biomarkers specifically for ERCC1, but also for BRCA1, p53R2, and TUBB3.

A comparison of K-M survival curves obtained using IHC and qRT-PCR methodologies showed that there was the same trend for all the biomarkers except for TUBB3 the tendency of which was inverted showing that patients with lower TUBB3-mRNA expression levels had a better OS. In addition, a significant correlation between protein and transcript was observed for RRM1 and TS (*r* = 0.29 and *r* = 0.47, resp.) and for TS and TUBB3 (*r* = −0.27) only. These findings are in agreement with preliminary evidence reported by others [67], which showed that there is not always a correlation between mRNA and protein expression levels. The lack of significant correlation between transcript and protein observed for most of the biomarkers could be explained by several causes. Our study is a retrospective study with an uncontrolled patient selection process that could potentially generate conflicting data. In addition, the use of archived material for the analysis of gene and protein expression has some limitations including the quantity and quality of available tissue and RNA degradation. Moreover, we should also take account of some aspects of PCR. The technique of RT-PCR allows for establishing quantitative mRNA expression profile of cells and tissues for which the sequence of the genes is known. However, the mRNA expression patterns are necessary but are by themselves insufficient for the quantitative description of biological systems. These evidences include discoveries of posttranscriptional mechanisms controlling the protein translation rate [68], the half-lives of specific proteins or mRNAs [69], and the intracellular location and molecular association of the protein products of expressed genes [70]. Moreover, due to the tumour heterogeneity, also IHC analysis with the use of TMA is limited, based on the quality and quantity of the tissue, being the small biopsies collected not necessarily representative of the whole extension of malignant disease. For the same reason, the intratumour heterogeneous biomarkers distribution and expression within the tumours may have influenced our results [46]. Altogether, these aspects could explain the reason for the lack of correlation between gene and protein expression levels and between biomarkers mRNA expression and OS observed in our study.

Finally, we decided to compare the results obtained by qRT-PCR with those obtained by the analysis of the largest NSCLC microarray dataset [5] available and used as a reference in many studies that includes high quality clinical and pathological data. A substantial agreement was observed between the K-M survival plots for our patients with the corresponding K-M survival plots for the microarray dataset. We observed a concordance in both the direction and the size of the effect of gene expression on OS for all biomarkers with the exception of ERCC1 indicating that the result obtained for ERCC1 is specific of our set of patients. The NSCLC microarray dataset confirmed RRM2 and TS as markers significantly associated with OS in univariate analysis; multivariate analysis showed TS as an independent marker of OS, supporting its prognostic potential in NSCLC patients.

## 5. Conclusion

In summary, RRM2 and TS protein expressions were identified as unfavourable prognostic markers in curatively resected NSCLC patients. Patients whose tumours are positive for RRM2 and TS have a significantly worse survival with a twofold increased adjusted hazard in patients with NSCLC overexpressing these markers. This information could be useful to select patients that might be treated with adjuvant chemotherapy. The mechanism behind the reduced survival in these patients warrants further research and has yet to be elucidated by* in vitro* research and by specifically designed, prospective studies that address the prognostic role of RRM2 and TS expression in NSCLC resected patients.

## Figures and Tables

**Table 1:** Patients and tumours characteristics.

Characteristics	Number
Number of patients	82
Median age at diagnosis (Y, median range)	69 (47–32)
Gender	
Female	20 (24%)
Male	62 (76%)
Smoking habit	
Never-smokers	6 (7%)
Ex-smokers	22 (27%)
Smokers	54 (66%)
Histological type	
Adenocarcinoma	50 (61%)
Large cell carcinoma	3 (4%)
Squamous cell carcinoma	28 (34%)
Other	1 (1%)
Pathological stage	
IA	23 (28%)
IB	21 (26%)
IIA	4 (5%)
IIB	11 (13%)
IIIA	16 (20%)
IIIB	7 (8%)
Surgery	
Bilobectomy	11 (13%)
Lobectomy	70 (85%)
Pneumonectomy	1 (1%)

**Table 2:** PCR primer sequences.

Gene		Accession number	Primer	Amplicon length
B2M	*Beta*-2-microglobulin	NM_004048	Frw TGA CTT TGT CAC AGC CCA AG	113
			Rev AGC AAG CAA GCA GAA TTT GG	

GUSB	*Beta*-glucuronidase	NM_000181	Frw GCC TGT GAC CTT TGT GAG C	109
			Rev GTG CCC GTA GTC GTG ATA CC	

BRCA1	Breast cancer 1, early onset	NM_007294	Frw TCA GCT TGA CAC AGG TTT GG	91
			Rev TCT GTA GCC CAT ACT TTG GAT G	

ERCC1	Excision repair cross-complementing rodent repair deficiency	NM_001166049	Frw AAT GTG CCC TGG GAA TTT G	104
			Rev TAG TCT GGG TGC AGG TTG TG	

RRM1	Ribonucleotide reductase subunit M1	NM_001033	Frw GAG GAA TTG GTG TTG CTG TG	97
			Rev ACT CTC AGC ATC GGT ACA AGG	

RRM2	Ribonucleotide reductase subunit M2	NM_001034	Frw TGA ACT GAA GAT GTG CCC TTA C	102
			Rev TTA CGG ACA ATT CAT GGT GTG	

P53R2	Ribonucleotide reductase subunit M2B	NM_001172477	Frw TCT CCC TCA CTG GAA CAA GC	130
			Rev AAC CTG CAC CTC CTG ACT AAA G	

TUBB3	Tubulin, *beta*-3	NM_001197181	Frw GAA GCC AGC AGT GTC TAA ACC	111
			Rev GGA GGA CGA GGC CAT AAA TAC	

TS	Thymidylate synthase	NM_001071	Frw CGG TGT GCC TTT CAA CAT C	105
			Rev TGT GCA TCT CCC AAA GTG TG	

**Table 3:** Patients characteristics, clinicopathologic features, and IHC analysis: association with OS. Results from Kaplan-Meier univariate and Cox multiple regression analyses.

Covariates	*N*	5-year survival			Kaplan-Meier			Cox regression^d^		
		Deaths		SP^*∗*^						
		*n*	%	%	HR	95% CI	*p* value^b^	HR	95% CI	*p* value^e^
Gender							0.967			0.611
Female	20	9	45.0	54.6	1			1		
Male	62	28	45.2	54.2	1.12	0.51–2.47		1.28	0.50–3.30	
Age at diagnosis							0.850			0.619
≤70 years	46	20	43.5	56.5	1			1		
>70 years	36	17	47.2	50.6	1.06	0.55–2.03		1.22	0.56–2.66	
Smoking habit							0.066			0.091
Ex/never-smoker	28	9	32.1	67.2	1			1		
Current smoker	54	28	51.9	47.7	1.99	0.94–4.24		1.98	0.90–4.39	
Pathological TNM							<0.001^c^			<0.001
Stage I	44	12	27.3	71.9	1			1		
Stage II	15	9	60.0	40.0	2.81	1.18–6.69		3.51	1.35–9.07	
Stage III	23	16	69.7	30.4	4.44	2.08–9.46		6.54	2.86–14.93	
Histological type							0.998			0.857
Nonsquamous	54	25	46.3	52.9	1	—		1		
Squamous	28	12	42.9	56.9	0.97	0.49–1.93		0.92	0.37–2.31	
ERCC1							0.126			0.059
≤140	33	12	26.4	61.4	1			1		
>140	49	25	51.0	47.5	1.70	0.85–3.39		2.08	0.97–4.44	
BRCA1^a^							0.308			0.750
Negative	36	14	40.9	60.8	1			1		
Positive	45	22	60.0	46.7	1.42	0.72–2.77		1.14	0.51–2.54	
TS^a^							0.242			0.023
Negative	66	27	40.9	58.8	1			1		
Positive	15	9	60.0	40.0	1.56	0.74–3.32		2.93	1.16–7.42	
p53R2^a^							0.297			0.699
Negative	57	27	47.4	52.1	1			1		
Positive	24	9	37.5	61.9	0.67	0.32–1.43		0.70	0.27–1.83	
TUBB3 (median *H*-score)							0.152			0.354
≤60	42	22	52.4	46.8	1			1		
>60	40	15	37.5	62.3	0.62	0.32–1.19		0.68	0.30–1.55	
RRM1^a^ (median *H*-score)							0.832			0.624
≤120	43	19	44.2	54.7	1			1		
>120	38	17	44.7	55.3	1.07	0.56–2.07		0.82	0.36–1.84	
RRM2 (median *H*-score)							0.069			0.031
≤140^a^	42	16	35.7	61.9	1			1		
>140	39	21	53.8	45.9	1.84	0.95–3.56		2.26	1.08–4.74	

^*∗*^SP = cumulative probability of surviving, ^a^expression level missing for 1 subject, ^b^log-rank (Mantel-Cox) test, ^c^test for trend, ^d^Cox regression analysis performed on 81 subjects with complete data: HR and 95% CI estimated from the final regression model for covariates retained in the model and from the full model for variables removed, and ^e^likelihood ratio test *p* value.

**Table 4:** Correlation between protein and mRNA expression levels.

Gene	Correlation^a^	BRCA1	ERCC1	TS	RRM1	RRM2	TUBB3
BRCA1	*r*	−0.040	0.188	−0.060	−0.009	−0.083	−0.032
	Number	80	80	79	79	80	80

ERCC1	*r*		0.152	−0.040	0.078	0.092	−0.080
	Number		82	81	81	82	82

TS	*r*			0.467^*∗∗*^	0.137	0.046	−0.270^*∗*^
	Number			77	77	78	78

RRM1	*r*				0.286^*∗*^	0.094	−0.034
	Number				71	71	71

RRM2	*r*					0.063	−0.111
	Number					75	75

TUBB3	*r*						−0.114
	Number						80

^a^Pearson's correlation coefficient, ^*∗∗*^ *p* value < 0.01, and ^*∗*^ *p* value < 0.05. Analysis was performed on subjects with complete data (range between 71 and 82).

**Table 5:** Patients characteristics, clinicopathological features, and RT-PCR analysis: association with OS. Results from Kaplan-Meier univariate and Cox multiple regression analyses performed on the subset of patients with complete data (*N* = 64).

Covariates	*N*	5-year survival			Kaplan-Meier			Cox regression		
		Deaths		SP^*∗*^						
		*n*	%	%	HR	95% CI	*p* value^a^	HR^c^	95% CI	*p* value^d^
Gender							0.872			0.826
Female	15	6	60.0	59.3	1			1		
Male	49	21	43.9	56.4	1.07	0.43–2.67		0.68	0.26–1.78	
Age at diagnosis							0.922			0.678
≤70 years	38	16	42.10	57.9	1			1		
>70 years	26	11	42.3	53.6	0.96	0.45–2.08		0.77	0.27–2.19	
Smoking habit							0.125			0.174
Ex/never-smoker	23	7	30.4	69.0	1			1		
Current smoker	41	21	50.0	49.3	1.97	0.83–4.66		1.81	0.76–4.30	
Pathological TNM							<0.000^b^			0.001
Stage I	37	9	24.3	74.5	1			1		
Stage II	11	7	65.6	36.4	3.49	1.30–9.34		3.49	1.30–9.40	
Stage III	16	11	78.7	31.3	4.39	1.81–10.7		4.39	1.41–10.68	
Histological type							0.519			0.777
Nonsquamous	39	18	46.2	52.5	1			1		
Squamous	25	9	46.0	63.7	0.77	0.35–1.71		0.79	0.29–2.11	
ERCC1 (median mRNA expression level)							0.574			0.619
<0.419	38	15	39.5	60.4	1			1		
≥0.419	26	12	47.2	52.7	1.24	0.58–2.66		1.14	0.38–3.44	
BRCA1^a^ (median mRNA expression level)							0.970			0.854
<0.071	36	15	41.7	57.8	1			1		
≥0.071	28	12	42.9	56.7	1.02	0.47–2.17		0.97	0.37–2.55	
TS^a^ (median mRNA expression level)							0.264			0.554
<0.179	28	10	35.7	63.7	1			1		
≥0.179	36	17	47.2	52.1	1.56	0.71–3.41		1.21	0.48–3.08	
p53R2 (median mRNA expression level)							0.915			0.807
<0.054	34	14	42.2	58.4	1			1		
<0.054	30	13	42.7	55.7	1.04	0.49–2.28		1.07	0.39–2.93	
TUBB3 (median mRNA expression level)							0.408			0.355
<0.453	33	12	46.4	63.6	1			1		
≥0.453	31	15	48.4	50.2	1.38	0.65–2.95		1.46	0.67–3.18	
RRM1							0.255			0.293
≤0.128	31	11	45.5	64.4	1			1		
>0.128	33	16	49.50	50.6	1.56	0.73–3.37		1.63	0.75–3.54	
RRM2							0.209			0.228
<0.275	31	11	45.5	64.0	1			1		
≥0.275	33	16	49.5	50.1	1.64	0.76–3.53		1.2	0.42–3.46	

^*∗*^SP = cumulative probability of surviving, ^a^log-rank (Mantel-Cox) test, ^b^test for trend, ^c^Cox regression analysis performed on 64 subjects with complete data: HR and 95% CI estimated from the final regression model for covariates retained in the model and from the full model for variables removed, and ^d^likelihood ratio test *p* value.

**Table 6:** Results from the DCC NSCLC microarray subset analyses.

Covariates	*N*	5-year survival			Kaplan-Meier			Cox regression^c^		
		Deaths		SP^*∗*^						
		*n*	%	%	HR	95% CI	*p* value^a^	HR^c^	95% CI	*p* value^d^
Gender							0.036			0.199
Males	174	74	52.7	55.1	1			1		
Females	156	47	47.3	66.8	0.68	0.47–0.98		1.25	0.87–1.80	
Age at diagnosis	330	121	36.7	0.43	1.03	1.01–1.04	0.008	1.03	1.01–1.05	0.003
Smoking habits							0.224			0.747
Ex/never-smoker	216	71	65.5	63.9	1			1		
Current smoker	25	10	7.6	56.2	1.32	0.68–2.57		0.86	0.56–1.33	
Unknown	89	40	26.9	53.6	1.39	0.94–2.05		1.09	0.53–2.26	
Pathological TNM							0.000^b^			0.000
Stage I	232	60	25.9	71.2	1			1		
Stage II	84	49	58.3	39.4	3.19	2.19–4.66		3.32	2.15–4.62	
Stage III	14	12	85.7	0.00	6.45	3.43–12.15		5.39	2.84–10.23	
ERCC1 (median mRNA expression level)							0.241			0.525
≤−0.045	181	68	37.6	0.60	1			1		
>−0.045	149	53	35.6	0.61	0.96	0.67–1.37		1.39	0.96–2.04	
BRCA1 (median mRNA expression level)							0.010			0.614
≤0.048	137	40	29.2	0.68	1			1		
>0.048	193	81	46.7	0.53	1.64	1.12–2.39		0.95	0.58–1.56	
TS (median mRNA expression level)							0.003			0.017
≤−0.018	166	48	38.1	0.67	1			1		
>−0.018	164	73	44.5	0.59	1.78	1.24–2.56		1.57	1.08–2.28	
TUBB3 (median mRNA expression level)							0.395			0.890
≤−0.116	189	63	33.3	0.63	1			1		
>−0.116	141	58	41.1	0.57	1.17	0.82–1.67		1.21	0.82–1.78	
RRM1 (median mRNA expression level)							0.130			0.419
≤−0.130	194	65	33.5	0.63	1			1		
>−0.130	136	56	41.2	0.57	1.32	0.92–1.89		0.88	0.59–1.32	
RRM2 (median mRNA expression level)							0.005			0.757
≤0.213	155	46	29.7	0.67	1			1		
>0.213	175	75	42.9	0.55	1.69	1.17–2.44		1.06	0.59–1.88	

^*∗*^SP = cumulative probability of surviving, ^a^log-rank (Mantel-Cox) test, ^b^test for trend, ^c^HR and 95% CI from the final regression model for covariates retained in the model and from the full model for variables removed, and ^d^likelihood ratio test *p* value.
